# Putative molecular mechanism underlying sperm chromatin remodelling is regulated by reproductive hormones

**DOI:** 10.1186/1868-7083-4-23

**Published:** 2012-12-17

**Authors:** Manjeet Kaur Gill-Sharma, Jyoti Choudhuri, Mukhtar Aleem Ansari, Serena D’Souza

**Affiliations:** 1 Department of Neuroendocrinology, National Institute for Research in Reproductive Health, J.M. Street, Parel, Mumbai 400012, India; 2 Junior Research Fellow (DST), Department of Neuroendocrinology, National Institute for Research in Reproductive Health, J.M. Street, Parel, Mumbai 400012, India; 3 College of Medicine, King Faisal University, Hofuf, Kingdom of Saudi Arabia; 4 Technical Officer, EM Laboratory, Department of Toxicology, National Institute for Research in Reproductive Health, J.M. Street, Parel, Mumbai 400012, India

**Keywords:** Reproductive hormones, Chromatin remodelling, Molecular mechanism, Histones, Spermiogenesis

## Abstract

**Background:**

The putative regulatory role of the male reproductive hormones in the molecular mechanism underlying chromatin condensation remains poorly understood. In the past decade, we developed two adult male rat models wherein functional deficits of testosterone or FSH, produced after treatments with 20 mg/Kg/d of cyproterone acetate (CPA) per os, for a period of 15 days or 3 mg/Kg/d of fluphenazine decanoate (FD) subcutaneously, for a period of 60 days, respectively, affected the rate of sperm chromatin decondensation in vitro. These rat models have been used in the current study in order to delineate the putative roles of testosterone and FSH in the molecular mechanism underlying remodelling of sperm chromatin.

**Results:**

We report that deficits of both testosterone and FSH affected the turnover of polyubiquitylated histones and led to their accumulation in the testis. Functional deficits of testosterone reduced expression of MIWI, the 5-methyl cap binding RNA-binding protein (PIWIlike murine homologue of the Drosophila protein PIWI/P-element induced wimpy testis) containing a PAZ/Piwi-Argonaut-Zwille domain and levels of histone deacetylase1 (HDAC1), ubiquitin ligating enzyme (URE-B1/E3), 20S proteasome α1 concomitant with reduced expression of ubiquitin activating enzyme (ube1), conjugating enzyme (ube2d2), chromodomain Y like protein (cdyl), bromodomain testis specific protein (brdt), hdac6 (histone deacetylase6), androgen-dependent homeobox placentae embryonic protein (pem/RhoX5), histones h2b and th3 (testis-specific h3). Functional deficits of FSH reduced the expression of cdyl and brdt genes in the testis, affected turnover of ubiquitylated histones, stalled the physiological DNA repair mechanism and culminated in spermiation of DNA damaged sperm.

**Conclusions:**

We aver that deficits of both testosterone and FSH differentially affected the process of sperm chromatin remodelling through subtle changes in the ‘chromatin condensation transcriptome and proteome’, thereby stalling the replacement of ‘dynamic’ histones with ‘inert’ protamines, and altering the epigenetic state of condensed sperm chromatin. The inappropriately condensed chromatin affected the sperm chromatin cytoarchitecture, evident from subtle ultrastructural changes in the nuclei of immature caput epididymal sperm of CPA- or FD-treated rats, incubated *in vitro* with dithiothreitol.

## Background

Sperm nuclear chromatin is remodelled during mid-spermiogenesis in the rat testis [1,2]. Nuclear chromatin condensation is characterised by the displacement of somatic histones H4//H3/H2A/H2B in step9-12 elongating spermatids of stages IX-XII seminiferous tubules of rat [3]. The core histones are replaced with testicular histone variants, deemed to condense chromatin and protect sensitive regions like telomeres/centromeres/imprinting clusters [4-6]. Histones are sequentially displaced by transition proteins, which are eventually replaced by arginine- and cysteine-rich protamines that remodel the nucleosomal chromatin solenoids into a novel torroidal conformation, which fundamentally changes its epigenetic state [7,8]. Condensed chromatin, which is heavily methylated, entrains formation of both endonuclease-sensitive as well as -resistant regions, depending upon its methylome that could involve certain DNA binding molecules for maintaining its stability and architecture, while protecting the DNA from damage, degeneration, chromosomal translocations and mutations [9,10]. However, a complement of testicular variants of somatic histones remains associated with torroidal chromatin where it likely condenses/protects sensitive DNA regions as well as determines the cytoarchitecture of the condensed chromosomes [11,12]. The cytoarchitecture of remodelled chromosomes, involving matrix associated messenger RNAs, within the sperm nuclei, could be important for their sequential de-condensation and subsequent activation of embryonic genes in the fertilised embryos [13-15].

The displacement of histones from chromatin solenoids during spermiogenesis and subsequent disassembly of nucleosomes appears to be rather complex involving sequential acetylations of H4 core histone at lysines (K) 5, 8, 16 with K12 being acetylated specifically in step9 elongating rat spermatids [16,17]. CDYL, H4 histone acetyltransferase activity and histone deacetylase1 (HDAC1), co-expressed specifically in step9-12 elongating murine spermatids, permit the hyperacetylation of H4 histones [17-19]. H4, di-acetylated at lysines K5K8 positions, interacts with a single bromodomain (BD1) of double testis-specific, bromodomain (BRDT) which facilitates its displacement [20-22]. Hyperacetylated H4 (H4ac), observed to co-exist with DNA strand breaks in step9-11 elongating murine spermatids, is not co-localised to nicked DNA [23,24]. Rather, the induction of physiological strand breaks in the DNA of step9-11 elongating murine spermatids correlated well with the co-incident expression of topoisomeraseIIβ (TOPIIβ) and tyrosyl DNA phisphodiesterase1 enzymes that induce as well resolve TOPIIβ-mediated DNA nicks as well as γ-H2AX, marker of double strand breaks [25]. H2A acetylated at lysine5 position (H2AK5), acetylated H2B and H3 have also been demonstrated in step9-11 murine elongating spermatids [17]. That H3 di-acetylation at positions 9/14 is necessary for the condensation of chromatin, became evident in Pygopus2 mutant mice, which are infertile [26].

H4 acetylated histones are polyubiquitylated and degraded in the 26S proteasomes of elongating spermatids. Polyubiquitylated histones H2A and H3 have been identified in step9-12 elongating rat spermatids whereas 20S proteasome subunits, in the range of 25-31Kd molecular mass, have been reported to be present in step13-15 condensing spermatids wherein the replacement of histones by transition proteins is complete [27,28]. The enzymes involved in ubiquitylating histones viz ubiquitin activating enzyme (E1), ubiquitin conjugating enzyme (E2) and ubiquitin ligating enzyme (E3) are expressed in the testis [29-33]. Whereas monoubiquitylation of H2A and H2B has been implicated in gene repression, polyubiquitylation of H4 is necessary for nucleosome disassembly [34]. The disruption of murine ubiquitin ligase gene (RNF8), which also induces H4 acetylation of lysine at position 16 (H4K16), has been reported to lead to defects in disassembly of nucleosomes during spermiogenesis [35]. The turnover of polyubiquitylated histones is regulated by the polyubiquitin-associated-zinc-finger domain (PAZ) of histone acetylase6 (HDAC6) and its deubiquitylating chaperone, p97 segregase (Valosin containing protein/VCP), co-expressed in elongating spermatids [36,37]. The molecular mechanism of sperm chromatin condensation, involving a histone to protamine transition, appears to have been conserved, since an identical mechanism has been reported in Drosophila melanogaster testis [38-40].

The regulatory role of reproductive hormones is well established in rodent spermatogenesis [41-43]. However, the putative roles of testosterone and follicle stimulating hormone (FSH), in the molecular events underlying the process of chromatin remodelling, remains to be elucidated in rat. We have used our indigenous, testosterone or FSH deficient rat models to study the effects of functional deficits of reproductive hormones on the histone to protamine transition *in vivo*, in order to elucidate the putative roles of reproductive hormones in regulating the molecular mechanism integral to chromatin remodelling during spermiogenesis [44-46].

## Results

### Reproductive hormone deficits adversely affect histone levels in maturing spermatids and epididymal sperm nuclear ultrastructure

**Figure 1:**
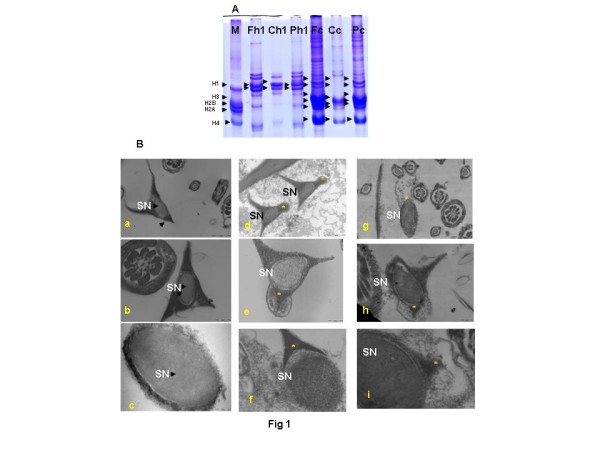
**(A) Representative electrophoretogram showing coomassie stained histone bands in the rat testis, separated by 15% Acid Urea Polyacrylamide Gel Electrophoresis.** Lane M: Calf thymus core somatic histone standard (H1 21.5Kd; H2A 14Kd; H2B 13.8Kd; H3 15.3Kd; H4 11.3Kd); Lane Fh1: H1 histones in FD-treated samples; Lane Ch1: H1 histones in control samples; Lane Ph1: H1 histones in CPA-treated samples; Lane Fc: Core histones in FD treated samples; Lane Cc: Core histones in control samples; Lane Pc: Core histones in CPA treated samples. Arrows indicate histone bands in standard and samples. (**B**) Representative ultramicrographs of immature caput epididymal rat sperm heads, treated in vitro with dithiothreitol (DTT). Left panel: Ultramicrographs of sperm heads of control rats at: (a) 30,000; (b) 68,000; (c) 68,000 magnification showing normal nuclei. Middle panel: Ultramicrographs of rat sperm heads of CPA-treated rats at: (d) 30,000; (e) 68,000; (f) 68,000 magnification showing subtle nuclear defects. Right panel: Ultramicrographs of rat sperm heads of FD-treated rats at: (g) 30,000; (h) 49,000; (i) 68,000 magnification showing subtle nuclear defects. (→) Arrows indicate immature epididymal sperm nuclei with condensed chromatin and normal perforatoria. (_*_) indicates immature epididymal sperm nuclei with loosely packaged chromatin and abnormal perforatoria with distended plasmalemma. SN, sperm nucleus.

Basic proteins extracted from testis of control, fluphenazine decanoate (FD) and cyproterone acetate (CPA)-treated rats were resolved on continuous 15% acid urea polyacrylamide gels. Basic protein bands were stained with coomassie blue (Figure 1A). Analysis of the electrophoretogram revealed that core histones H2A, H2B, H3 and H4, identified by comparison with standard calf thymus histone bands, persisted at unusually high levels in the testis of rodents deficient in either testosterone or FSH. Ultramicrographs of sperm heads taken from the caput region of the epididymides of control (Figure 1B a-c), CPA-treated (Figure 1B d-f) and FD-treated (Figure 1B g-i) rats, treated with dithiothreitol *in vitro*, demonstrated nuclear defects, namely swollen, deformed, ruptured nuclei with abnormal perforatoria and distended plasma membrane. It is evident from the presence of sperm with chromatin condensation defects in the caput epididymides of CPA- and FD-treated rats, that functional deficits of testosterone or FSH have adversely affected the process of chromatin condensation in the maturating elongating spermatids in at least some of the seminiferous tubules. Since the CPA and FD treatments were for short durations, it was more relevant to look for qualitative rather than quantitative effects.

### Testosterone deficits adversely affect the process of histone acetylations in maturing spermatids in rat testes

**Figure 2:**
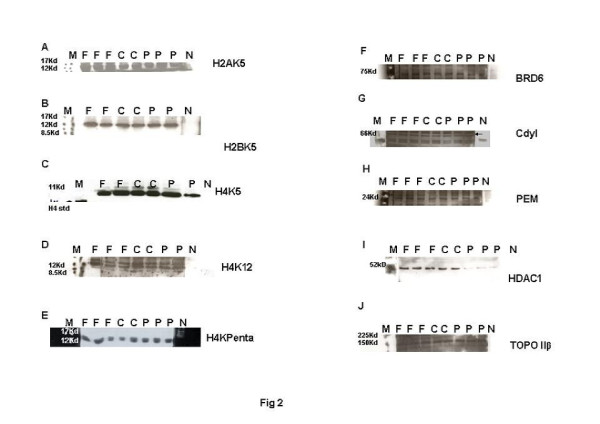
**Representative electrophoretogram of rat testis showing bands of acetylated core histones and enzymatic molecules implicated in displacement of nuclear histones during sperm chromatin remodelling. **(**A**) H2AK5. (**B**) H2BK5. (**C**) H4K5. (**D**) H4K12. (**E**) H4Kpenta. (**F**) BRD6. (**G**) HDAC1. (**H**) Cdyl. (**I**) TOPIIβ. (**J**) PEM. Lane M: Rainbow markers. Lane F: FD-treated samples. Lane C: Control samples. Lane P: CPA-treated samples. Lane N: Negative controls.

**Figure 3:**
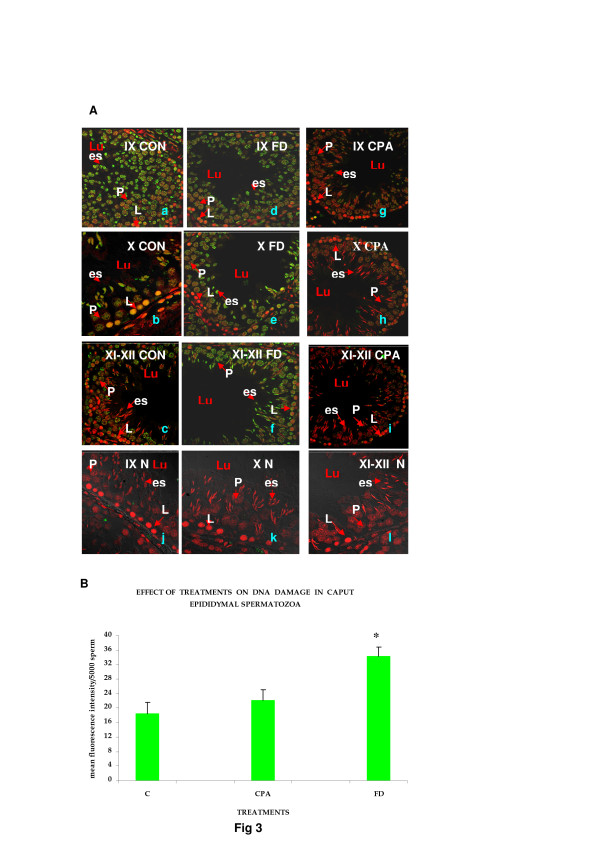
**(A) Representative confocal images of rat testis wax sections (2 μ) at 882 magnification, showing dUTP-FITC incorporation in nicked (green) and PI nuclear counterstain (red) in the DNA of elongating rat spermatids of stages IX to XII seminiferous tubules. **Left panel-CON: Control. Middle panel-FD: FD-treated. Right panel-CPA: CPA-treated. N: Negative controls. a/d/g/j: stage IX seminiferous tubule. b/e/h/k: stage X seminiferous tubule. c/f/i/l: stages XI to XII seminiferous tubules. Lu: Lumen. Arrows indicate: P: Pachytene spermatocytes. L: Leptotene spermatocytes. es: elongating spermatids. (**B**) Flow cytometric analysis of DNA integrity in rat sperm taken from caput epididymides. Histogram showing dUTP-FITC incorporation in nicked DNA strands. C: Control samples. FD: FD-treated samples. CPA: CPA-treated samples. (*) indicates a significant difference.

**Figure 4:**
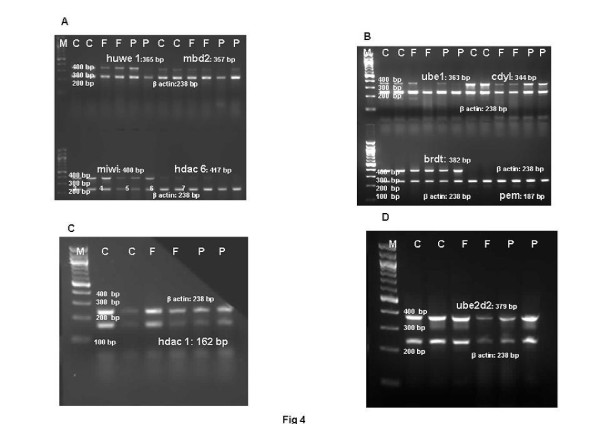
**Representative electrophoretogram of rat testis showing bands of genes implicated in histone replacement during chromatin remodelling. **(**A**) *huwe1, miwi, hdac 6, mbd2. *(**B**) *ube1, brdt, cdyl, pem. *(**C**) hdac. (**D**) *ube2d2. *Lane M: NEB 100 bp Ladder. Lane C: Control samples. Lane F: FD-treated samples. Lane P: CPA-treated samples.

**Figure 5:**
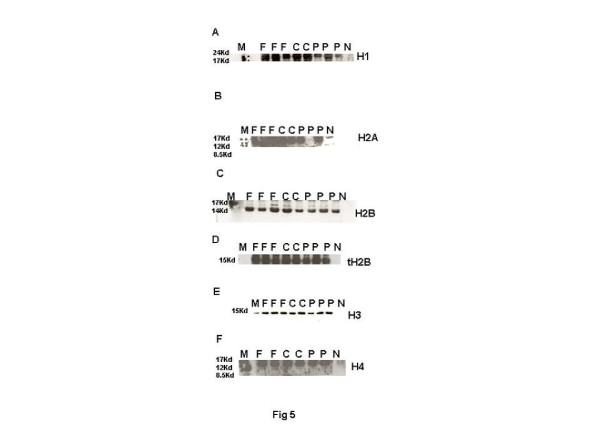
**Representative electrophoretogram of rat testis showing bands of linker and core histones. **(**A**) H1. (**B**) H2A. (**C**) H2B. (D) tH2B. (**E**) H3. (**F**) H4. Lane M: Rainbow markers. Lane F: FD-treated samples. Lane C: Control samples. Lane P: CPA-treated samples. Lane N: Negative controls.

**Figure 6:**
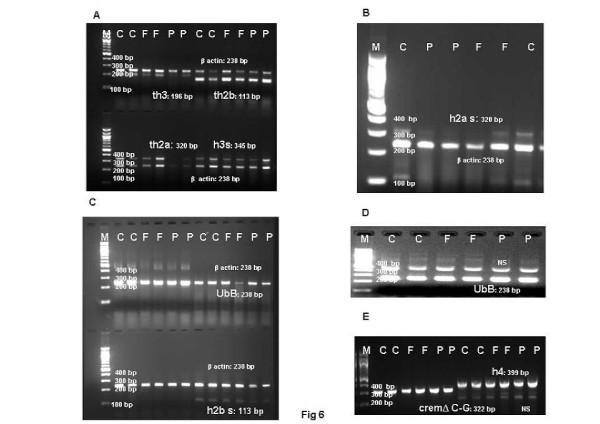
**Representative electrophoretogram of rat testis showing bands of histones, ubiqutin, cremΔC-G genes implicated in sperm chromatin remodelling.** (**A**) *th3, h3s, th2a, th2b. *(**B**) *h2as.* (**C**) *h2bs.* (**D**) *ubB* (Ubiquitin). (**E**) *h4, cremΔC-G*. Lane M: NEB 100 bp Ladder. Lane C: Control samples. Lane F: FD-treated samples. Lane P: CPA-treated samples. NS: non-specific bands.

Histone to protamine exchange involves acetylation of spermatidal histones at several positions. In view of the results indicative of stalled process during chromatin remodelling, basic proteins were extracted from the rat testis and resolved on polyacrylamide gels. Modified histones were then detected with specific antibodies. No overt changes were detectable in the levels of H4K5, H4K12, H2AK5, H2BK5, H4 penta-acetylated histones on the electrophoretograms of CPA-treated rat testis (Figures 2A-E). Levels of BRD6 and CDYL were also not affected in testosterone-deficient rats (Figures 2F-2G). The levels of PEM (Rhox5), a control androgen-dependent protein, were also not overtly affected (Figure 2H). However, a virtual knockout of histone deacetylase1 (HDAC1) enzyme was evident in the testis of testosterone-deficient rats (Figure 2I). In view of the loss of HDAC1 and the role of sequential histone acetylations in histone to protamine transition, the process of nucleosomal disassembly was also investigated in the rat testis. Abundance of FITC fluorescence in the elongating spermatids of stage IX seminiferous tubules of control rat testis indicated the presence of characteristic DNA single/double strand breaks that occur to relieve torsional stress and facilitate nucleosomal disassembly (Figure 3Aa). Abundance of PI fluorescence and absence of FITC in the elongating spermatids of stage IX seminiferous tubules indicated the absence of DNA single/double strand breaks in CPA-treated rats (Figure 3Ag). Gradual disappearance of FITC and abundance of PI fluorescence in the elongating spermatids of stages X to XII seminiferous tubules of control rats indicated normal physiological repair of DNA strand breaks (Figure 3Ab and c). Persistence of PI and absence of FITC fluorescence in the elongating spermatids of stages X to XII seminiferous tubules was suggestive of failure of the DNA repair mechanism in the CPA-treated rats (Figure 3Ah and i). Results of the qualitative TUNEL assay clearly suggested that functional deficiency of testosterone affected the process of nucleosomal disassembly in particular. Interestingly, quantitative TUNEL assay indicated the absence of nicked DNA in the immature caput epididymal sperm nuclei of CPA-treated rats (Figure 3B). In view of the absence of nicked DNA in the testis of testosterone-deficient rats, the levels of topoisomeraseIIβ (TOPIIβ), implicated in the physiological process of induction of DNA strand breaks to relax chromatin during nucleosomal disassembly, were ascertained by western blotting on polyacrylamide gels. Type II topoisomerases are nuclear matrix proteins expressed in spermatids which change DNA topology by catalysing single or double stranded breaks to relieve torsional stress. The electrophoretogram did not reveal any change in the levels of TOPIIβ (Figure 2J). Mbd2 gene has been implicated in maintaining the chromatin architecture in rat testis. The expression of mbd2 gene was downregulated in the testis of CPA-treated rats (Figure 4A). No changes could be detected in the levels of acetylation candidates, namely somatic variants of core histones linker histone H1, H2A, H2B, H3, H4, or testis-variant tH2B by western blotting on polyacrylamide gels (Figures 5A-F). However, testosterone deficiency quantitatively reduced the expression of several genes implicated in histone to protamine exchange, in the testis of CPA-treated rats as compared to control rats, namely somatic cdyl, brdt, pem, hdac1, th3, h2b (Figures 4B-C, Figure 6A, Figure 6C; Table 1). Murine PIWIli or MIWI, a chromatoid body RNA-binding protein that associates with 5-methyl messenger RNA cap on polysomes, has been implicated in post-transcriptional regulation of stabilised messenger RNAs stored in cytoplasmic chromatoid bodies. The testicular expression of miwi gene was downregulated in the testis of CPA treated rats as compared to control rats (Figure 4A).

**Table 1:** Expression of chromatin condensing genes in FD/CPA-treated rat testis

**Rat genes**	**PCR product (bp)**	**Ratio**		
		**(Mean ± S.D**.) **= IOD of specific gene/ β Actin**		
		**Control**	**FD**	**CPA**
*ube1)*	363	0.271 ± 0.03	0.124 ± 0.06	0.079 ± 0.02^a^
*ube2d2*	379	1.261 ± 0.016	1.324 ± 0.052	1.564 ± 0.07^a^
*huwe1(e3)*	365	0.41 ± 0.087	0.35 ± 0.04	0.33 ± 0.16
*hdac6*	417	0.13 ± 0.003	0.108 ± 0.01	0.061 ± 0.001^a^
*ubB*	238	-(monoplex)	N	N
*cdyl*	344	0.824 ± 0.02	0.246 ± 0.009^a^	0.602 ± 0.002^a^
*brdt*	382	1.826 ± 0.03	1.43 ± 0.018^a^	1.40 ± 0.02^a^
*hdac1*	162	0.668 ± 0.062	0.53 ± 0.13	0.41 ± 0.05^a^
*miwi/piwi*	400	1.115 ± 0.003	0.92 ± 0.25	0.925 ± 0.02^a^
*mbd2*	357	0.13 ± 0.01	0.109 ± 0.007	0.073 ± 0.01^a^
*pem*	187	0.0154 ± 0.0006	0.02 ± 0.003	0.0015 ± 0.0003^a^
*cremΔC-G*	322	-(monoplex)	N	N
*h2a somatic*	320	0.131 ± 0.01	0.17 ± 0.0009	0.05 ± 0.005^a^
*h2b somatic*	113	0.094 ± 0.005	0.099 ± 0.003	0.059 ± 0.007^a^
*h3 somatic*	345	0.549 ± 0.07	0.37 ± 0.03	0.47 ± 0.18
*th2a*	320	0.872 ± 0.053	0.877 ± 0.06	0.712 ± 0.002
*th2b*	113	3.4426 ± 0.66	3.11 ± 0.73	3.25 ± 0.217
*h3t*	196	0.428 ± 0.012	0.266 ± 0.12	0.22 ± 0.036^a^
*h4*	399	-(monoplex)	N	N
*Mouse actin*	238	-	-	-

IOD of PCR product bands is a mean of three observations, quantified with Gel Pro 3.1 image analysis software.

Details of primers given in Table 3.

^a^Significant difference.

N, No differences seen in monoplex RTPCR.

### Testosterone deficits adversely affect the ubiquitination of histones in maturing spermatids in rat testis

**Figure 7:**
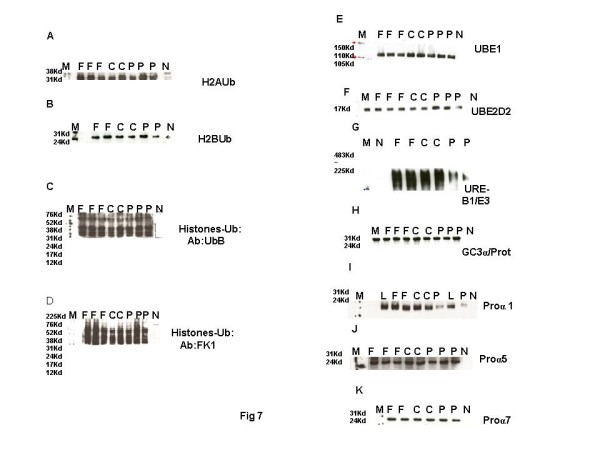
**Representative electrophoretogram of rat testis showing bands of ubiquitylated histones and enzymatic molecules implicated in histone ubiquitination during chromatin remodelling. **(**A**) H2AUb. (**B**) H2BUb. (**C**) UbHistones probed with Ubiquitin antibody. (**D**) UbHistones probed with FK1 antibody. (**E**) UBE1. (**F**) UBE2D2. (**G**) URE-B1/E3. (**H**) GC3α. (**I**) Proα1. (**J**) Proα5. (**K**) Proα7. Lane M: Rainbow markers. Lane F: FD-treated samples. Lane C: Control samples. Lane P: CPA-treated samples. Lane N: Negative controls. L: HeLa cell lysate (Positive control).

In view of the observed persistence of histones in the testis of CPA-treated rats, levels and expression of ubiquitylated histones, ubiquitylating enzymes UBE1, UBE2D, URE-B1/E3, hdac6, proteasomes α1, α5, α7 and HDAC6 were ascertained in the rat testis. Histones, their ubiquitylating enzymes and ubiquitinating proteasomes were extracted, resolved on polyacrylamide gels, and detected with specific antibodies. No overt changes were detected in the testicular levels of ubiquitylated H2A or H2B on the electrophoretograms (Figures 7A, 7B). However, accumulation of total ubiquitylated histones was observed on the electrophoretogram of CPA-treated rats, when probed with anti-ubiquitin ubB antibody (Figure 7C). More importantly, accumulation of polyubiquitylated histones was observed when CPA-treated testicular proteins were probed with FK1 antibody, raised to polyubiquitylated lysozyme at lysine 48 through formation of intra-ubiquitin isopeptide bonds (Figure 7D). FK1 antibody was incapable of detecting ubiquitin or monoubiquitylated histones. The expression of testicular ubiquitin itself appeared to be unaffected (Figure 6D). A decrease in the levels of ubiquitin ligating enzyme URE-B1/E3 and reduced expressions of hdac6 involved in promoting ubiquitination, Ube1, Ube2d2, but not huwe1/active domain of ure-b1/e3, was observed on the electrophoretograms of CPA-treated rats (Figure 7G; Figure 4A, B and D; Table 1). Simultaneously, the levels of testicular 20S proteasome α1, but not proteasomes α5 and α7, were also observed to decrease on the electrophoretograms of CPA-treated rats (Figure 7I-K).

### FSH deficits adversely affect the process of histone acetylations and chromatin repair in maturing spermatids in rat testes

In view of the accumulation of histones at high levels in the testis of FD-treated rats, basic proteins were extracted from the rat testis, separated on polyacrylamide gels and modified histones detected by western blotting. No changes were seen in the levels of H4K5, H4K12, H2AK5, H2BK5, H4 penta-acetylated histones, BRD6, HDAC1, CDYL, TOPIIβ, PEM (Rhox5) on the electrophoretograms of FD-treated rat testis (Figures 2A-G). The levels of acetylation substrates, namely somatic variants of core histones linker histone H1, H2A, H2B, H3, H4, or testis-variant tH2B, seen by western blotting on polyacrylamide gels, were also unaffected (Figures 5A-F). The expression of genes implicated in chromatin condensation, namely mbd2, miwi, pem, hdac1, somatic th3, tH2a, th2b, h3s, h2as, h2bs, ubB, h4, cremΔC-G, was not affected (Figures 4A-C, Figures 6A-E; Table 1). However, the expression of cdyl and brdt genes was reduced in FD-treated rat testis (Figure 4B). The failure of the process of DNA repair during nucleosomal disassembly was also evident from the abundance of FITC fluorescence in the qualitative TUNEL assay, indicating persistence of nicked DNA, in the elongating spermatids of stages XI to XII seminiferous tubules of FD-treated rats (Figure 3Ad and e) as compared to control rats (Figure 3Ab and c). Quantitative TUNEL assay also detected a significant increase in immature sperm with nicked DNA in the caput region of the epididymides of FD-treated rats as compared to control rats (Figure 3B).

### FSH deficits affect the process of histone ubiquitination in maturing spermatids in rat testis

Since histones persisted in the testis of FD-treated rats, histones and their ubiquitylating enzymes were extracted, separated on polyacrylamide gels, and detected with specific antibodies. Testicular levels of ubiquitylated H2A or H2B were not affected on the electrophoretograms (Figures 7A, 7B). However, total ubiquitylated histones increased on the electrophoretogram of FD-treated rat testis, when probed with anti-ubiquitin histone antibody ubB and FK1 antibody specific to polyubiquitylated histones, whereas the expression of ubiquitin was unaffected (Figure 7C, 7D, Figure 6D). The levels of UBE1, UBE2D2, URE-B1/E3, Pro α1, Pro α5 and Pro α7, on the electrophoretograms of testis of FD treated rats, were also not affected (Figure 7E-K). The expressions of Ube1, Ube2d2 and huwe1/e3 too were unaffected on the electrophoretograms of testis of FD-treated rats (Figure 4A, B and D; Table 1). Interestingly, the expression of hdac6, implicated in the turnover of ubiquitylated histones due to its polyubiquitin binding PAZ domain, was also unaffected in testis of FD-treated rats (Figure 4A).

## Discussion

It is well established that sperm chromatin remodelling in rat is initiated in the nuclei of elongating spermatids of stage IX seminiferous tubules. The remodelling process essentially involves sequential acetylations of core nuclear histones. Hyperacetylated histones are important for the relaxation of chromatin supercoiled in solenoids by recruiting factors capable of inducing and repairing transient nicks in DNA strands. Acetylated histones are polyubiquitylated and degraded in the proteasomes of elongating spermatids in stages XII to XIV seminiferous tubules, followed by their sequential replacement with transition proteins and protamines. The appearance of transition proteins correlates with the condensation of chromatin whereas protamines remodel it into a novel torroidal conformation. Whereas normal condensation of chromatin is essential for the development of the embryo, chromatin condensation defects are associated with abnormal nuclear development, as has been observed in transition protein1, transition protein2, protamine2 and FSH receptor knockout mice [47,48]. Persistence of mono-ubiquitylated histones has also been observed in sperm nuclei of FSHRKO mice [49]. CPA- and FD-treated rat models, with functional deficits of testosterone or FSH, pre-established in the past decade in our laboratory, have been used in the current study, to elucidate the regulatory roles of testosterone and FSH, in the molecular mechanism underlying histone displacement, integral to the process of chromatin condensation [44-46]. We had previously reported that CPA or FSH treatments, with functional deficits of testosterone and FSH, increased the rate of *in vitro* decondensation of mature epididymal sperm chromatin. This effect was attributed to the considerable reduction in protamine deposition, and persistence of histones, at unusually high levels, in the epididymal sperm, several of which had overt abnormalities in their perforatoria [50]. These results had indicated that sperm that had matured in a testicular milieu functionally deficient in either testosterone or FSH had chromatin condensation defects. The results were suggestive of a hormone-dependent deregulation of the mechanism underlying the turnover of ubiquitylated histones, crucial for histone to protamine transition during spermiogenesis. Based on the results of our previous studies, which had demonstrated that reproductive hormones affect chromatin remodelling, we have further used these hormone-deficient rat models to ascertain the roles of testosterone and FSH in the molecular mechanism underlying the histone to protamine exchange, integral to the condensation of sperm chromatin. The expression of genes involved in histone to protamine exchange occurs in the round spermatids stage VII seminiferous tubules. Conventionally, stabilised transcripts involved in spermiogenesis are purportedly stored in cytoplasmic chromatoid bodies adjacent to the spermatidal nuclei and translated later in appropriate stages [51,52]. The chromatin of the elongating spermatids of stage IX seminiferous tubules is transcriptionally repressed. The transcriptional and translational processes are thus rendered independent of each other with the result that protein levels may not always be correlated to transcript levels in the testes [53]. We have therefore quantified the expression of those molecules that have been implicated in the mechanism of histone modifications and displacement and degradation during chromatin remodelling, in the testis of these rat models.

We have previously reported that cyproterone acetate, which has the dual property of being a progestational antiandrogen, blocks the androgen receptors, expressed uniquely in the Sertoli cells in the rat testis, while balancing the release of pituitary gonadotropins and maintaining peripheral levels of testosterone in CPA-treated rat model [45,54,55]. Currently, functional deficiency of testosterone was re-confirmed in the testis of CPA-treated rats from the downregulated expression of androgen-dependent pem/rhox5 gene, expressing a transcription factor implicated in the regulation of androgen-dependent genes in the Sertoli cells [56,57]. Downregulated expression of another androgen-dependent, androgen-binding protein gene had been reported previously, in the testis of CPA-treated rats [45].

We now report that histones accumulate at high levels, in the testis of rats deficient in testosterone. However, the testicular levels of nuclear histones H1, H2A, H2B, H3 and H4 were unaffected in CPA-treated rat testis. Testicular expression of nuclear histones th2a, th2b, h2as, h3s, h4, with the exception of h2bs, th3, was, however, unaffected [3-6,58]. Abnormal persistence of the tH2B, concomitant with protamine deficits, has been reported in the sperm of infertile human males [59,60]. It is thus tempting to suggest that modified tH2B and H3 histones could have accumulated in the testis of CPA-treated rats as a consequence of downregulation of h2bs and th3 genes. It is therefore inferred that testosterone could be playing a role in the expression of h2b and th3 genes.

Nuclear histones undergo seqential acetylations at lysines at several positions which facilitate their displacement. No overt changes were detectable in the testicular levels of H4K5, H4K12, H2AK5, H2BK5, H4 penta-acetylated histones. Nevertheless, a decrease observed in the expression of cdyl and brdt genes, though not protein levels, suggested a role for testosterone in the molecular mechanism underlying histone acetylations and displacement [21]. This role was further substantiated from the downregulated expression and levels of HDAC1 enzyme, purported to be involved in maintaining the status of histone acetylation, in CPA-treated testis [17,18,25].

Sequential acetylations of histone H4 facilitate their displacement and determine the state of relaxation of chromatin. The stability of chromatin is also purportedly determined by MBD2 protein, which binds methylated cytosines in DNA to secure the transposons and imprinted genes, and maintains chromatin architecture. We have observed a decrease in the expression of testis-specific mbd2 gene in the CPA-treated rat testis suggestive of an altered state of chromatin supercoiling resistant to relaxation [61]. Relaxation of supercoiled chromatin in the elongating spermatids of stage IX seminiferous epithelium depends upon the appearance of topoisomerase TOPIIβ enzyme-mediated DNA strand breaks [24]. We neither observed the expected DNA nicks in the stage IX elongating spermatids nor did reduction in TOPIIβ levels in the testis of CPA-treated rats. It is therefore averred that factors other than TOPIIβ could be responsible for stalling the physiological process of nicking DNA, which facilitate chromatin relaxing in the elongating stage IX spermatids. Thus inappropriate acetylations and displacement of histones could have affected the purported recruitment of factors, capable of inducing transient nicks in highly compacted DNA strands, which facilitate relaxation of supercoiled DNA, namely γ-H2AX (marker histone of double strand nicks), tyrosyl-DNA phosphodiesterase1 known to both create as well as resolve topoisomerase-mediated DNA nicks, poly(ADP-ribose)polymerases1, 2, which catalyse the poly(ADPribosy)lation of histones, deemed to reduce their affinity for DNA [62-64]. Thus, the absence of some androgen-dependent factors affected the molecular mechanism of histone displacement in the elongating spermatids of CPA-treated rats.

Polyubiquitylation at a specific lysine in histones is necessary for their degradation. We have observed accumulation of total and ubiquitylated histones in the testis of CPA-treated rats, suggestive of a stalled process of clearance. Whereas levels of ubiquitylated H2A and H2B were unaffected, the expression and/ levels of huwe1 (active region of ure-b1/e3) and hdac6, implicated in the ubiquitylation and subsequent transport of histones, as well as levels of 20S proteasome α1, involved in their degradation, were reduced in the testis of CPA-treated rats. The expression/levels of enzymes ube1, ube2d2, huwe1 (active region of URE-B1/E3) involved in uiquitin ligation, 20S proteasomes α5, and α7, as well as ubiquitinB were unaffected. It is therefore averred that functional testosterone insufficiency primarily reduced the ubiquitylation of histones, their transport and degradation during histone to protamine exchange [28,31,33,36,37].

Histones, that have not been degraded, persist in the nuclei of the elongated spermatids and lead to the formation of sperm with abnormal nuclear shapes [50]. When mature caput sperm of CPA-treated rats were treated with DTT *in vitro*, ultrastructural studies demonstrated swollen nuclei which had burst as compared to control sperm, indicative of the presence of an unusual conformation of chromatin. We had previously reported the presence of high levels of histones in these sperm [50]. We have currently observed that the testicular expression of MIWI, an RNA-binding protein, implicated in the post-transcriptional inhibition of protein translation, is downregulated in the testes of CPA-treated rats. MIWI has been implicated in the expression of certain mi (micro) and pi (MIWI-dependent PIWI interacting) RNAs, necessary for the inhibition of translation on polysomes. It is tempting to link the downregulation of MIWI to the losses of HDAC1, URE-B1/E3 and 20S proteasome α1 and consequent accumulation of modified histones, in particular that of ubiquitylated tetis-specific tH2B and polyubiquitylated somatic H3, in the testis as well as mature sperm of CPA treated rats [51,52,65]. It is averred that histone displacement was incomplete after CPA treatment and that functional testosterone deficits interfered with the molecular mechanism of sperm chromatin remodelling.

FSH, on the contrary, appears to be playing a different yet complex role, in tandem with that of testosterone, in remodelling chromatin during spermiogenesis. We have earlier shown that treatment with fluphenazine decanoate, which blocks the dopamine receptors in the pituitary gland, produced moderate hyperprolactinemia, which effectively reduced the peripheral levels of LH and FSH while maintaining those of testosterone, in rats [55]. In the current studies, histones were observed to accumulate at high levels in the testis of FSH-deficient rats. However, testicular levels of histones H1, H2A, H2B, tH2B, H3, H4, expression of tH2A, tH2b, tH3, h2as, h2bs, h3, the levels of acetylated histones, namely H4K5, H4K12, H2AK5, H2BK5, H4 penta-acetylated histones were unaffected in FD-treated rats. The level and expression of HDAC1 did not change in the testis of FSH-deficient rats. Albeit, expression, of cdyl and brdt genes was reduced but not levels, as compared to the normal expression of the control cremΔC-G gene [66]. The results clearly suggested a co-regulatory role for FSH in the mechanism of sequential acetylation and displacement of histones [17,18,21,24,25]. *A priori*, normal testicular levels of TOPIIβ and the presence of nicked DNA in the elongating spermatids of stage IX seminiferous tubules, suggested that molecules involved in nicking DNA were not FSH-dependent [53,67,68]. However, the persistence of nicked DNA throughout stages IX to XII seminiferous tubules, as well as significant increase of nicked chromatin in the pool of mature caput sperm of FD-treated rats, indicated the failure of a FSH-mediated chromatin repair mechanism, operative in the elongating spermatids of stages XI to XII tubules [63,67]. It is therefore averred that FSH-mediated histone acetylations, occurring at specific sites during the relaxation of DNA, may be involved in the recruitment of crucial DNA repair proteins, namely transition protein1, p53, DNA polymerase [24,50,69,70]. We had reported in the past the loss of at least one repair protein, namely transition protein1 in the testis of FD-treated rats [44]. Interestingly, literature reports implicate MIWI in the regulation of FSH-dependent cremτ and its target genes [71]. However, in the current study, the expression of miwi gene was unaffected in the testis of FD-treated rats [51]. Intriguingly, literature reports suggest that the loss of miwi and cremτ gene products have overlapping effects indicative of a common mechanism of post-transcriptional gene regulation in the chromatoid body [52]. It is, therefore, tempting to suggest that availability of K1F17b kinesin, a testis-specific, nucleo-cytoplasmic mRNA shuttle, purportedly required for the post-transcriptional regulation of transition protein1, implicated in a DNA repair mechanism specific to elongating spermatids, was affected by the loss of cyclic AMP response modulator protein (CREMτ) observed in our previous studies [72]. Albeit, downregulation of the cdyl and brdt genes observed in the present study, is speculated to have occurred due to reduced availability of putative FSH-dependent miRNAs involved in their post-transcriptional regulation [52,65].

Apart from defective acetylation, accumulation of core as well as ubiquitylated histones was observed in the testis, suggesting that the process of histone ubiquitination was stalled in FD-treated rats. Testicular levels of ubiquitylated histones H2A and H2B, though, were unaffected. Testicular levels of UBE1, UBE2D2, URE-B1, 20S proteasomes α1, α5 and α7, enzymes of the ubiquitinating complex too were not affected by fluphenazine treatment. However, in our previous studies, we had observed that histones persisted at high levels in the nuclei of the elongated spermatids, which led to the formation of sperm with abnormal perforatoria in the epididymides of FD-treated rats [50]. Current studies have also shown ultrastructural defects in the nuclei of decondensed caput sperm indicative of the presence of an unusual conformation of chromatin. It thus became evident that core/ubiquitylated histones, persisting in the testis of FSH-deficient rats, could be responsible for an altered chromatin conformation. Our studies suggested that molecules involved in the turnover of ubiquitylated histones could be playing an important role in chromatin condensation. The expression of testis-specific, deubiquitylating enzymes (DUBs) in the elongating spermatids, namely USP2 (UBP testis), p97/VCP corroborate this opinion [73]. Recruitment of repair proteins to the site of radiation-induced, DNA lesions has been reported for RNF8 ubiquitin ligase-p97 segregase in Homo sapiens [74]. It is therefore speculated that FSH deficits may have affected the levels of p97 segregase, and stalled the mechanisms of turnover of ubiquitylated histones and DNA repair, particularly since the expression of hdac6 which binds polyubiquitylated proteins and promotes their turnover was not affected [36,37]. It is tempting to suggest that p97 deficits could be involved in the stalled mechanisms of ubiquitination and chromatin repair in the stages XI to XII elongating spermatids in the testis of FD-treated rats.

Chromatin condensation entails profound cytoarchitectural changes in sperm chromatin without any change in the structure of the genes. Basically, replacement of dynamic histones, which determine the epigenetic state of chromatin, with inert protamines is responsible for the profound change in the epigenetic status of sperm chromatin. The epigenetic regulation of gene expression is dependent upon the chromatin methylome as well as the presence of histones which actually recruit nuclear transcription regulatory factors. If the process of histone displacement is stalled due to subtle changes in the chromatin remodelling transcriptome and proteome, it can lead to an altered epigenetic status. In the present study, functional deficits of testosterone and FSH produced subtle effects on the chromatin remodelling transcriptome and proteome. Reduced expression of crucial genes involved in the biological process of chromatin remodelling profoundly affected the cytoarchitecture of epididymal sperm chromatin. Inappropriately condensed chromatin may fail to decondense at a species specific rate upon fertilization and lead to inappropriate expression of early genes involved in embryogenesis.

## Conclusions

Testosterone and FSH impact a common chromatin remodelling mechanism during spermiogenesis through chronological expression of different molecules required for this biological process to take place. All our results suggest that the molecular mechanism underlying histone to protamine exchange is dependent upon testosterone for the expression of genes involved in the relaxation of supercoiled chromatin solenoids whereas FSH is essential for the expression of genes required for repair of relaxed chromatin in elongating spermatids. Androgen receptor polymorphisms/mutations and endemic stress generated by fluctuating peripheral prolactin levels, prevalent across the globe are potential causes for the occurrence of chromatin condensation defects associated with male infertility. As such, additional studies to identify all the modified histones that persist in the testis/sperm, using a sensitive mass spectroscopic technique, would be beneficial in understanding the aetiology of chromatin condensation defects associated with male infertility [75]. Further studies to localise testicular variants of molecules involved in the molecular mechanism of histone replacement during chromatin remodelling would be fruitful in understanding the genesis of defective chromatin condensation. In view of our observation that a molecular mechanism for the removal of defective elongating spermatids does not exist in the testis, studies designed to detect changes in sperm chromatin methylome would help gain insights into the aetiology of congenital defects in embryos/resorptions [76].

## Methods

### Pharmacological interventions

Male Holtzmann virgin rats, aged 2.5 months, were administered CPA (Androcur, Schering, Berlin, Germany) suspended in water by sonication, at a dose of 20 mg/kg/d per os, for a period of 15 days in order to induce functional androgen deficiency as previously described [45]. Age-matched control rats were administered water. Male Holtzmann virgin rats, aged 2.5 months, were administered FD (Prolinate, Sun Pharmaceuticals, India) dissolved in sesame oil, at a dose of 3 mg/kg/d s.c, for a period of 60 days in order to induce selective FSH deficiency as previously described [46]. Age-matched control rats were administered sesame oil. The study protocol was approved by the Institutional Animal Ethics Committee (IAEC) of National Institute for Research in Reproductive Health, Parel, Mumbai 400012, India (Registration No. 78/1999/CPCSEA dated 11/3/1999).

### Collection of biological samples

Some batches of rats were sacrificed by cervical dislocation after the last treatment, testis were dissected out, rinsed in hyposaline (4.5 g/L) to remove traces of blood and either stored at −80°C for western analyses or immersion fixed in 10% neutral buffered formalin, dehydrated through a series of graded ethanol (30% to 100%) and processed for paraffin wax embedding for routine histology. Paraffin sections were used for the analysis of DNA damage. Testis from all three experimental batches were also immediately frozen in liquid nitrogen and stored at −80°C for molecular genetic studies. Some batches of anaesthetised rats from all three groups were perfused with phosphosaline 0.01 M containing heparin (5,000 IU/L), in order to clear blood from the epididymides, before releasing sperm from the caput region for analysing DNA damage.

### Electrophoretic separation of basic testicular proteins by acid urea PAGE

Basic proteins were extracted as previously described [50]. A total of 500 mg of testis tissue were homogenised in 9 mL of cold 0.5 M HCl and incubated on ice for 20 min. Supernatants were collected by centrifugation at 8,000 rpm at 4°C for 10 min. Basic proteins were precipitated out of the supernatants with trichloroacetic acid, at a final concentration of 20%. Protein pellets obtained by centrifugation at 10,000 rpm for 10 min at 4°C, were washed thrice with chilled acetone, air dried, dissolved in 0.5% glacial acetic acid containing 5 M urea and 5% βmercaptoethanol and stored at −20°C.

### Extraction of linker Histone H1

A total of 1.5 g of testis from each rat were homogenised in 5% perchloric acid and centrifuged at 8,000 rpm for 20 min at 4°C. Histone H1 was precipitated from supernatant with trichloroacetic acid, at a final concentration of 20%, and separated by centrifugation at 8,000 rpm for 20 min at 4°C. Precipitates were washed twice with acetone, air dried and dissolved in 0.1% glacial acetic acid containing 5% βmercaptoethanol and stored at −20°C. The concentrations of basic proteins/H1 in the samples were quantified spectroscopically from absorbance (O.D) at 280 nm (O.D of 1 = 1 mg/mL). Basic proteins were separated on 15% polyacrylamide gels by continuous acid urea polyacrylamide gel electrophoresis (AUPAGE) in the indigenous TechnoSource microkin electrophoresis apparatus (Mumbai, India) provided with bidirectional current flow circuit. Fifteen millilitres resolving gel mixture: 2.25 g urea extrapur (MP Biochemicals, USA), 30% acrylamide:bisacrylamide (29.7:0.3 g ratio), 0.75 mL glacial acetic acid, 1 mL DW, 45 μL 20% APS, 255 μL TEMED, was allowed to polymerise at room temperature (RT) for 1 h. A 10-well comb was inserted prior to polymerization. A total of 1 μL of 2% methyl green tracking dye in buffer (0.5% glacial acetic acid, 5 M urea, 5% βmercaptoethanol) was loaded in a reference well. The polyacrylamide gels were pre-electrophoresed at 25 to 30 mA constant current, from anode to cathode, with 5.1% acetic acid as electrophoresis buffer, till the dye ran off the gel (2.30 h). The wells were washed thoroughly to remove traces of urea with electrophoresis buffer with a syringe. The electrophoresis buffer in the lower tank was replaced with fresh buffer. Samples of 50 to 100 μg were directly loaded per well. A total of 2 μL calf thymus core histone (Worthington, USA)/ H1 standard (Roche, Germany) in buffer (0.5% acetic acid, 5 M urea, 5% βmercaptoethanol and 1uL methyl green tracking dye) was loaded in the reference well. Final electrophoresis was done at 20 mA constant current using 5.1% acetic acid as running buffer for 1 h at RT. Gel was stained with 0.125% coomassie blue (in 40% methanol, 7% glacial acetic acid and 53% DW) for 10 min at RT. Gels were destained in a solution of 40% methanol, 7% glacial acetic acid, 53% DW at RT. Coomassie stained protein bands were scanned for documentation. Core and linker histones bands in the calf thymus histone standard were used as reference to identify histones in the basic proteins.

### Electrophoretic separation of testicular histones by SDS PAGE for western blotting

Testicular histones were extracted by Sittman’s procedure as previously described [50]. Briefly, whole testis were thawed in 0.01 M PBS to remove tunica and allowed to stand on ice for 3 min in hyposaline to clear traces of blood, homogenised in 10 mM Tris–HCl buffer at pH7.2 containing 5 mM magnesium chloride, 0.5 mM phenylmethanesulfonyl fluoride (PMSF), 0.32 M Sucrose, 0.1% Triton X-100 and 1% βmercaptoethanol. Lysates were centrifuged at 5,000 rpm at 4°C for 5 min. Protein pellets were suspended in the same buffer and centrifuged as before. Protein pellets were re-suspended in 10 mM Tris–HCl buffer at pH7.2 containing 5 mM magnesium chloride, 0.5 mM PMSF, 0.25 M potassium chloride and incubated on ice for 20 min. Protein pellets were separated by centrifugation at 10,000 rpm at 4°C for 10 min, suspended in 0.2 M sulfuric acid and incubated on ice for 20 min. Supernatants obtained by centrifugation at 10,000 rpm for 10 min at 4°C were neutralised with 100% ammonium hydroxide solution and histones precipitated out with 100% chilled ethanol. Protein pellets obtained by centrifugation at 10,000 rpm at 4°C for 10 min were washed thrice with 50% ethanol, air dried, and dissolved in 0.1% glacial acetic acid containing 0.1% β-mercaptoethanol and kept at 4°C. Supernatants obtained after centrifugation at 5,000 rpm for 5 min at 4°C were stored at −20°C till further analysis by discontinuous SDS (sodium dodecyl sulphate) PAGE. For 10/12.5/15% polyacrylamide gels of 1.5 mm thickness, 10 mL resolving gel mixtures containing 3.3/4.16/5 mL of a 30% acrylamide:bisacrylamide (29.7:0.3 ratio), 2.5 mL of 1.5 M Tris buffer pH 8.9, 0.1 mL of 10% SDS, 4/3.13/2.3 mL of DW, 0.1 mL of 10% APS, 10 μL TEMED, overlayered with 1 mL DW were allowed to polymerise in the microkin electrophoresis for 50 min at RT. A 3 mL mixture of 5% stacking gel mixtures, containing 0.5 mL of 30% acrylamide:bis acrylamide (29.7:0.3 ratio), 0.38 mL of 1 M Tris base buffer pH 6.8, 30 μL of 10% SDS, 2.1 mL DW, 30 μL of 10% APS, 3 μL TEMED, was allowed to polymerise onto the resolving gels, inserted with a 10-well comb, for 60 min at RT. Sittman’s histone extracts (10 to 20 μg), mixed with an equal volume of 2X sample loading buffer (5 mL of spacer gel buffer diluted 1:10, containing 20% βmercaptoethanol, 20% glycerol, 2% SDS, 1 pinch bromophenol blue), were loaded per well without boiling. Loading order was interchanged in some of the gels in order to override the problem of incomplete protein transfer and incorrect interpretation, since it was observed that even slight changes in temperature or buffer composition led to uneven transfer. Rainbow markers (Amersham, UK), in the ranges of 3.5-38Kd/12-225Kd, were loaded in the reference well, in preference to calf thymus standard, because commercial anti-histone antibodies cross-reacted with only rat antigens and detected proteins of specific molecular masses. Protein bands in the Rainbow markers corresponding to the mass of the histones, were easily visible after transfer onto blotting membranes, and could be spotted with HRP-labelled secondary antibody for detection with ECL reagents. H1, H2As, H2Bs, H3, H4, tH2A, tH2B, tH3, H2AUb (ubiquitylated), H2BUb (ubiquitylated), Ub (ubiquitylated) histones, H2AK5 (acetylated on Lys 5), H2BK5 (acetylated on Lys 5), H4K5 (acetylated on Lys 5), H4K12 (acetylated on Lys12), H4Penta (acetylated on Lys 5, 8, 12, 16, 20) were separated on 10% to 15% polyacrylamide gels. Electrophoresis was done at 20 mA constant current, from cathode to anode for 50 min at RT with conventional SDS PAGE electrode buffer (25 mM Tris base, 192 mM Glycine, 0.1% SDS) ) as described by Laemmeli [77]. The gels were rinsed for 15 min in Singleton’s transfer buffer (5.81 g Tris base, 2.92 g glycine, 400 mg SDS, 20% methanol per litre). Histones were transferred onto 0.22 μ (0.45 μ for polyubiuitylated histones) nitrocellulose membranes with Singleton’s buffer at 50 V for 90 min (35 V for 3 h for H2bUb) at 4°C [60]. Complete protein transfer was confirmed with PonceauS stain. Membranes were destained with DW and blocked with 5% non-fat dry milk (NFDM) in phosphosaline 0.01 M containing 0.1% Tween-20 (PBST) for 1 h at RT, with moderate rocking. Membranes were incubated with affinity purified, anti-rat (cross-reacting) primary antibodies diluted in block buffer, overnight at 4°C (Table 2). Negative control strips were incubated in block buffer overnight at 4°C. H2AUb and polybiquitylated histones were incubated with primary IgM antibodies (Millipore/Enzo) diluted in PBS. Membranes were rinsed thrice with PBST for 10 min each, with vigorous rocking, and incubated with appropriate horseradish peroxidise (HRP)-conjugated secondary antibodies (Sigma/Santa Cruz), at appropriate dilutions, in 1% block buffer for 90 min at RT with moderate rocking. H2AUb, polyubiquitylated histones and negative control strips were incubated with anti-IgM biotinylated secondary antibody (Sigma), diluted in 0.01 M PBS, for 90 min at RT, with moderate rocking. Membranes were rinsed with PBST for 4 × 10 min, with vigorous rocking. Anti-IgM biotinylated secondary antibodies (for H2AUb/polyubiquitylated histones) were labelled with biotinylated HRP by incubating with avidin biotin complex for 1 h at RT in the dark without agitation (ABC was prepared according to Elite Universal Vector Lab kit protocol in 0.01MPBS, 30 min prior to use and incubated at 4°C). Membranes were finally rinsed with PBST for 4 × 10 min, with vigorous rocking. Strips with Rainbow marker bands were cut away after transfer, blot dried overnight, and spotted with 1 μL of HRP-conjugated anti-mouse secondary antibody (DAKO) at 1:100/200/400 dilution in 0.1 M phosphate buffer 10 min prior to detection. Histones were detected with ECL plus reagents as per Amersham kit protocol. X-Ray films (Amersham) were developed after 30 s to 15 min exposure to the blots (depending upon the signal intensity) in order to visualise chemiluminiscent signals.

**Table 2:** Details of primary and secondary antibodies used during western blotting

**Antibodies**	**MW (Kd)**	**Antibody dilutions**		
		**Primary**	**Secondary**	**Ref (primaries)**
UBE1 (N-20)	110	1:200 (Goat)	1:50,000 (Sigma)	Santa Cruz 47555
E217Kb/UBE2D2	17	1:1,000 (Rb)	1:50,000 (Sigma)	S.S.Wing (UBC4) (gift)
LASU1 /URE-B1/E3	485	1:1,000 (Rb)	1:50,000 (Sigma)	S.S.Wing (HUWE1) (gift)
CDYL (F-15)	66	1:50 (Goat)	1:20,000 (Sigma)	Santa Cruz 34147
BRDT/brd6	75	1:100 (Goat)	1:5,000 (s.c)	Everest EB05796
HDAC1(C-19)	52	1:100 (Goat)	1:50,000 (Sigma)	Santa Cruz 6298
TOPO IIβ (H-286)	180	1:200 (Rb)	1:5,000 (s.c)	Santa Cruz 13059
PEM (M-15)	21-24	1:50 (Goat)	1:4,000 (s.c)	Santa Cruz 21650
Proteasomes purified fish	24-31	1:3,000 (Ms Mab)	1:50,000 (Sigma)	T. Tokumoto (GC3α) (gift)
20S Proteasomeα1(C7)	24-31	1:200 (Ms Mab)	1:5,000 (s.c)	Santa Cruz 166073
20S Proteasomeα5(H-53)	24-31	1:250 (Rb)	1:5,000 (s.c)	Santa Cruz 67342
20S Proteasomeα7(MCP72)	24-31	1:500 (Ms MAb)	1:50,000 (Sigma)	Santa Cruz 58417
H1	17	1:20 (Ms Mab)	1:4,000 (s.c)	Abcam 62884
H2A somatic	14	1:20 (Rb)	1:20,000 (Sigma)	Biovision 3621-100
H2Bsomatic	14	1:3,000 (Rb)	1:4,000 (s.c)	Epitomics 1847-1
H3 somatic	15	1:1,000 (Rb)	1:50,000 (Sigma)	Santa Cruz 8654R
tH2B	15	1:250 (Rb)	1:25,000 (Sigma)	Millipore 07680
H4	11	1:20 (Rb)	1:20,000 (Sigma)	Biovision 3624-100
H2AUb	25	1:65 (Ms Mab/IgM)	1:8,500 (anti-IgM:Sigma)	Millipore 05678
H2BUb	25	1:100 (Mab)	1:4,000 (s.c)	Millipore 051312
Ub histones	12-225	1:50 (Rb)	1:4,000 (s.c.)	Abcam/UbB/7780
Ub histones FK1	12-225	1:100 (MsMab/IgM)	1:9,000 (anti-IgM:Sigma)	Enzo/FK1 PW8805
H2AK5	14	1:65 (Rb)	1:4,000 (s.c)	Cell Signalling 2576
H2BK5	14	1:200 (Rb)	1:4,000 (s.c)	Cell Signalling 2574
H4K5	11	1:5,000 (Rb MAb)	1:2,000 (s.c)	Epitomics 1808-1
H4K12	11	1:200 (Rb)	1:20,000 (Sigma)	Abcam 1761
		1:200 (Rb)	1:4,000 (s.c)	Cell Signalling 2591
H4K Penta	10	1:30 (Rb)	1:20,000 (Sigma)	Millipore 06946

~MW is denoted as Kd. GC3α MAb is used to detect Proteasomes. Ubiquitin antibody and FK1 MAb IgM antibody are used to detect ubiquitylated and polyubiquitylated histones respectively. H4 Penta is used to detect penta-acetylated H4.

### Electrophoretic separation of testicular non-histones by SDS PAGE for western blotting

Detunicated testes of 100 mg were homogenised manually on ice in 0.5 mL of RIPA buffer (Sigma) containing 0.5 mM PMSF or protease inhibitor cocktail (Sigma) or protease inhibitor (Amersham 1 tablet/10 mL buffer). The homogenates were centrifuged at 14,000 rpm at 4°C for 30 min and supernatants stored at −20°C till further western analysis. The protein concentration in RIPA extracts was quantitated by Lowry’s method [78]. For 5/7.5/10/12.5/15% polyacrylamide gels of 1.5 mm thickness, 10 mL resolving gel mixtures containing 1.67/2.5/3.3/4.16/5 mL of 30% acrylamide:bisacrylamide (29.2:0.8 ratio), 2.5 mL of 1.5 M Tris buffer pH 8.9, 0.1 mL of 10% SDS, 5.62/4.79/4/3.13/2.3 mL of DW, 0.1 mL of 10% APS, 10 μL TEMED, overlayered with 1 mL DW were allowed to polymerise in the microkin apparatus for 50 min to 2 h at RT. A 3/5 mL mixture of 5/4% stacking gel mixtures, containing 0.5/0.675 mL of 30% acrylamide:bis acrylamide (29.2:0.8 ratio), 0.38/0.625 mL of 1 M Tris base buffer pH 6.8, 30/50 μL of 10% SDS, 2.1/3.6 mL of DW, 30/50 μL of 10% APS, 3/5 μL of TEMED, were allowed to polymerise onto the resolving gels, inserted with a 10-well comb, for 60 min at RT. RIPA buffer protein extracts (50 to 100 μg), mixed with an equal volume of 2× sample loading buffer (5 mL of spacer gel buffer diluted 1:10, containing 20% βmercaptoethanol, 20% glycerol, 2% SDS, 1 pinch bromophenol blue), were boiled for 5 min, chilled, spun, and loaded per well. Loading order was interchanged in some of the gels in order to override the problem of incomplete protein transfer and incorrect interpretation, since it was observed that even slight changes in temperature or buffer composition led to uneven transfer. Rainbow markers in the range of 3.5-38Kd/12-225Kd were loaded in the reference well, in preference to beta actin, as specific loading controls were not commercially available for each of the sample proteins. Bands of specific molecular masses, corresponding to the mass detected by specific antibodies cross-reacting with rat antigens, were easily visible after protein transfer onto blotting membranes (unlike 42Kd beta actin) and could be used for chemiluminiscent detection. (URE-B1/E3 samples were not boiled and loaded in 4% stacking gels.) URE-B1/E3, UBE1, UBE2D2, URE-B1/E3, CDYL (chromodomainY like protein), BRDT (bromodomain testis-specific protein), HDAC1 (histone deacetylase), PEM (homeobox placentae and embryos oncofetal protein /Rhox5 reproductive homeobox5 protein), 20S proteasomes α1, α5, α7, TOPIIβ (topoisomerase), were separated on 5% to 15% gels by electrophoresing at 100 V constant voltage, for 75 min with conventional SDS PAGE electrode buffer (25 mM Tris Base, 192 mM Glycine, 0.1% SDS) as described by Laemmeli [77]. (HeLa cell lysate, gift from Santa Cruz, was loaded in one well as loading control on proteasome α1 gels.) The gels were rinsed for 15 min in conventional SDS PAGE Towbin’s transfer buffer (25 mM Tris base, 192 mM Glycine, 20% methanol). Proteins were transferred onto 0.22 μ/0.45 μ nitrocellulose membranes at 55 V for 90 min at 4°C with Towbin’s buffer [79]. (UBE1, URE-B1/E3 proteins were transferred onto 0.45 μ nitrocellulose membranes at 30 V overnight at 4°C). Complete protein transfer was confirmed with ponceau S stain. Membranes were destained with DW and blocked with 5% NFDM in phosphosaline 0.01 M containing 0.1% Tween-20 (PBST) for 1 h at RT. (Proteasomes were blocked with 0.5% gelatin (bacteriological, Glaxo, Mumbai, India) in PBST for 30 min at RT and then 5% NFDM-PBST for 60 min). Membranes were incubated overnight with primary antibodies at appropriate dilutions in block buffer at 4°C (Table 2). Negative controls were incubated in block buffer overnight at 4°C. Membranes were rinsed thrice with PBST for 10 min each, with vigorous rocking and incubated with appropriate HRP-conjugated secondary antibodies, at appropriate dilutions, in 1% block buffer for 1 h at RT with moderate rocking. Membranes were rinsed with PBST for 4 × 10 min, with vigorous rocking. Strips with Rainbow marker bands were cut away after transfer, blot dried overnight, and spotted with 1 μL of HRP-conjugated anti-mouse secondary antibody (DAKO, gift by Chetan, GIB lab, NIRRH) at a dilution of 1:100/200/400 in 0.1 M phosphosate buffer, 10 min prior to detection. Proteins were detected with ECL plus reagents as per Amersham kit protocol. Chemiluminiscent signals were visualised on a sensitive X-ray hyperfilm (28-9068-36-Amersham) after a 30 s to 15 min exposure depending upon signal intensity. Reference bands of a specific mass were used to detect specific antigens in the samples.

### Evaluation of sperm chromatin packaging by transmission electron microscopy

Immature sperm for the study of nuclear ultrastructure were released from cuts in the caput epididymides, into Dulbecco’s phosphosaline 0.01 M (without calcium/magnesium), by incubating for 60 min at 4°C. Sperm, pooled from caput epididymides of three rats in each treatment group, were pelleted by cold centrifugation at 2,000 rpm for 10 min, washed with cold hyposaline for 30 min to remove traces of blood and centrifuged as before. Final sperm pellets were incubated in 1 mL Dulbecco’s phosphosaline containing 10 mM dithiothreitol at 37°C for 60 min and centrifuged at 15,000 rpm for 10 min. Sperm pellets were washed twice with cold phosphosaline 0.01 M and hard pelleted by centrifugation at 4°C, at 15,000 rpm for 10 min. Pellets were fixed in cold 4% paraformaldehyde (containing 1% glutaraldehyde, 100 mg picric acid crystals, calcium chloride (12.5 mg) for 36 h at 4°C as previously described [50]. Fixative was removed and sperm were washed with 0.1 M cacodylate buffer by keeping on ice for 15 min, followed by centrifugation at 8,000 rpm for 8 min. Pellets were washed twice as above and post fixed in osmium tetraoxide and 0.2 M cacodylate buffer in 1:1 ratio at 4°C for 90 min. Osmium fixative was removed and sperm washed thrice with 0.1 M cacodylate buffer by keeping on ice for 15 min, followed by short spin and buffer was decanted. Pellets were transferred to fresh vials in 0.1 M cacodylate buffer. Immediately buffer was removed followed by addition of cold 30% ethanol on ice for 15 min. Procedure was repeated twice. Subsequently, pellets were washed twice with 50%, 70%, 80%, 90%, 95% dry acetone twice, as above. Finally, pellets were washed thrice with dry 100% acetone at 4°C, at RT, and once again at RT for 30 min. Acetone was removed and pellets allowed to be infiltrated with araldite, dodecenyl succinic anhydride (DDSA) in dry acetone in the ratio 1:1:2. Pellets were finally embedded in fresh araldite, DDSA and dimethyl aminomethyl phenol (DMP) at 55°C for 48 h, 60°C for 24 h. Blocks were stored at RT. Sections of 0.05 um thickness were stained with uranyl acetate and lead citrate. Several grids were scanned under the electron microscope for capturing images of sperm heads with overt nuclear defects, at three different magnifications. The effect was basically qualitative because only tubules at stages IX to XII of maturation were affected.

### Assessment of DNA strand breaks in seminiferous tubules in testis

The TUNEL (terminal nucleotidyl-mediated nick end labeling) assay for detecting the status of DNA damage in seminiferous tubules of rat testis, at different stages of chromatin condensation, has been done according to the method of Leduc *et al*. [24]. Testes were immersion fixed in freshly prepared 10% neutral buffered formalin and embedded in paraffin wax. Two micrometer sections were cut and placed on poly-Lysine coated slides. Slides were de-paraffinised by immersion in xylene for 15 min, once for 10 min, air dried, and cleared once in methanol for 10 min. Slides were hydrated twice for 5 min in distilled water and rinsed once in 0.01 M phosphosaline for 5 min. Slides were immersed in boiling solution of 10 mM Tris–HCl containing 1 mM ethylenediamine tetraacetate (EDTA) (pH 9) for 5 to 10 min. Slides were cooled in running tap water for 10 min. Area around the tissues were dried and marked with a hydrophobic pen. The tissues were permeabilised in phosphosaline containing 1.5% BSA and 0.5% Triton X-100 for 60 min at 37°C. Slides were rinsed by dipping thrice in phosphosaline, incubated with 100 uL of terminal deoxynucleotide transferase enzyme-FITC (fluorescein isothiocyanate) Roche *in situ* cell death detection kit mixture containing 0.1% Triton-X-100 in wells, in a humidified atmosphere for 60 min at 37°C in the dark. Enzyme was omitted in control slide wells according to kit protocol. Excess FITC was decanted at the end of incubation and slides rinsed once with phosphosaline. Slides were counterstained with 12.5 ng/50 uL of Propidium Iodide (PI) for 15 min at RT. Excess PI was decanted and slides rinsed twice with phosphosaline for 10 min each. The slides were mounted in antifade solution (Vectastain) and stored at 4°C overnight and scanned for staining under confocal microscope. Nicks in spermatidal DNA, where (deoxyuridine triphosphate) dUTP-FITC conjugate fluorochrome was incorporated at free hydroxyl ends, in stage IX to XII seminiferous tubules, were visualised from fluorescence under a confocal microscope at 520 nm. PI nuclear counterstain fluorescence was visualised at about 620 nm.

### Assessment of immature sperm DNA damage

The sperm from caput epididymides were released by incubating for 1 h at 37°C in 10 mL phosphosaline 0.01 M, diluted with 4 mL of phosphosaline and filtered through 40 μm cell strainers (BD Biosciences). Sperm in all samples were pelleted by centrifugation at 2,500 rpm for 10 min and fixed in 4% buffered formalin, added dropwise while vortexing. Sperm samples were fixed by placing on ice for 60 min. Fixed sperm samples were washed thrice with phosphosaline to remove traces of fixative and re-pelleted by centrifugation at 2,500 rpm for 10 min. Final pellets were dispersed in residual buffer in the tubes by tapping. Sperm were counted in a haemocytometer. Two million sperm/mL were suspended in 70% chilled ethanol added dropwise while vortexing in flow tubes and placed on ice for 30 min. Sperm samples were stored at −20°C overnight. Samples were centrifuged at 2,500 rpm for 10 min to remove ethanol by aspiration without disturbing the sperm pellets and processed for assessment of DNA damage according to TUNEL protocol (APO-DIRECT™ kit, BD Pharmingen). Briefly, sperm were suspended in 1 mL wash buffer 6548AZ added dropwise followed by 0.5 mL buffer to wash sides of tubes and allowed to stand at RT to disperse clumps. Sperm were pelleted by centrifugation as before. Wash step was repeated twice. Sperm were incubated in DNA labelling solution for 180 min in dark at 37°C in water bath. A total of 6 μL of 2.5 mM dithiothreitol was also added to labelling solution to a final volume of 51 μL. Terminal deoxynucleotide transferase (Tdt) enzyme was omitted from negative control tubes. At the end of incubations, sperm were rinsed twice with 1 mL of kit buffer 6550AZ by centrifugation at 2,500 rpm for 10 min. Sperm were suspended in 0.3 mL of kit PI/RNase staining buffer 6551AZ and incubated in dark for 60 min at RT. A total of 0.2 mL of phosphosaline was added to samples before filtering through 40 μm cell strainers. FITC fluorescence of 5,000 sperm measured immediately in a FACS Vantage SE flow cytometer equipped with an Argon laser. Results were expressed as mean ± sem. Significant differences between experimental and control group rats were calculated by Student’s t-test. Level of significance was set at *P* ≤0.05. In spite of heavy losses of sperm during the procedure, the increased fluorescence of nicked sperm, arising from affected seminiferous tubules only, could be quantified.

### Molecular genetic studies

Total RNA was extracted from individual samples of testis and specific transcripts amplified using gene specific primers, as previously described [45]. Briefly, 50 to 100 mg frozen testes tissues were detunicated and homogenised mechanically in 15 mL RNAse-free polypropylene tubes in 1 mL Trizol reagent and RNA extracted with 0.2 mL chloroform per mL. Lysates were allowed to stand for 5 min at RT and centrifuged at 12,000 g for 15 min at 4°C. Upper aqueous phases containing RNA were transferred to fresh tubes and RNA precipitated with 0.5 mL isopropanol per ml Trizol used at RT for 10 min. RNA was pelleted out by centrifugation at 12,000 g for 10 min at 4°C. RNA pellets were washed with 1 mL of chilled 70% ethanol, centrifuged at 7,500 g for 5 min, at 4°C and air dried. RNA pellets were solubilised in RNAse-free diethylpyrocarbonate (DEPC) treated water by warming at 65°C for 10 min and stored at −70°C for reverse transcriptase polymerase chain reaction (RTPCR). Concentration and purity of RNA was spectroscopically determined from the absorbance (O.D) of diluted samples (1:100) at 260 nm. RNA concentration in μg/μL was calculated as: 100 × 0.04 × O.D at 260 nm (0.04 μg/μL being RNA concentration at an O.D of 1 at 260 nm). RNA (0.5 to 1 μg) in individual samples was reverse transcribed at 48°C for 45 min using AMV reverse transcriptase and amplified by biplex polymerase chain reaction (PCR), according to titan one tube RTPCR kit protocol (Roche Diagnostics). Gene specific primers were used for the amplification of all mRNAs in a 50 μL reaction volume. Specific mouse βactin primers were used to amplify the control actin transcripts in the biplex PCR assay. The conditions of PCR used to amplify the genes for 35 cycles were: denaturation at 94°C × 2 min and 94°C × 30 s; annealing at 55°C × 1 min; extension for 68°C × 1 min; final extension at 68°C × 7 min. Gene specific primers for *ube1*, *ube2d2*, *ureb1*/*e3*, *cdyl*, *brdt*, *hdac1*, *hdac6*, *miwi* (RNA-binding protein in chromatoid body transports ACT (activator of cyclic AMP response element modulator-CREMτ), *mbd2* (methyl CpG binding protein), *pem*, *h2as* (somatic), *h2bs* (somatic), *h3s* (somatic), *th2a* (testis-specific), *th2b* (testis-specific), *th3* (testis-specific) were used for the amplification by biplex PCR as previously described (Invitrogen primer details in Table 3) [45]. Gene specific primers of *cremΔCG* (isoform of transcription factor CREM expressed during spermiogenesis, lacks exonsCG which express the phosphorylation domain and represses cyclic adenosyl monophosphate-induced transcription), *ubiquitinB and h4* could however detect the respective transcripts, only by monoplex PCR. PCR products (4 to 12 uL) in individual samples, containing 1 to 3 uL blue juice 10× loading dye, were resolved on 2% agarose gels (Horizon11-14 horizontal electrophoresis apparatus, Germany/TechnoSource mini horizontal electrophoresis apparatus, Mumbai, India), containing 0.5 ug/mL ethidium bromide with sterile 1XTAE (Tris-acetic acid-EDTA) running buffer, against 5 uL of markers (NEB). The resolved biplex PCR products, in individually extracted samples, were quantified with Gel Pro 3.1 software. mRNA concentration in samples was estimated from the ratios of IODs (integrated optical density) of specific PCR gene product and βactin control gene product. IOD ratios of biplex PCR take care of variations in extraction/amplification during the procedure. Ratios were expressed as mean ± S.D. (Table 1). Significant differences between experimental and control group rats were calculated by Student’s t-test. Level of significance was set at *P* ≤0.05.

**Table 3:** Details of PCR primers used for molecular genetic studies

**Rat genes**	**PCR product (bp)**	**PCR primers**	**Gene ID/Accession /GenBank number**
*ube1x*		Left primer: 5^′^-GAG-AGG-AAA-TGG-TTC-TCA-CAG-ATT-3^′^	
	363	Right primer: 5^′^-AAT-ACT-TGG-CAA-AGT-CAG-TCA-TCA-3^′^	314432
*E217Kb/*		Left primer: 5^′^-CAC-AGT-GGT-CTC-CAG-CAC-TAA-CTA-3^′^	
*ube2d2*	379	Right primer: 5^′^-GCT-AGG-AGA-CAG-TGT-TGG-TAC-AGA-3^′^	U56407
*huwe1 mouse fragment (ure-b1/e3)*		Left primer: 5^′^-GGA-GTA-TGT-GAA-GTT-CGT-GAC-CTC-3^′^	
	365	Right primer: 5^′^-GTA-CCT-GTG-ACA-AAC-TGG-AGG-AAC-3^′^	59026
*hdac6*		Left primer: 5^′^-ACA-ACT-GCA-CAG-TGT-GCT-TCAG-3^′^	
	417	Right primer: 5^′^-ACA-AGG-TTG-GGA-CAC-ATC-TAGG-3^′^	84581
*ubB*		Left primer: 5^′^-GTT-TGT-TCC-TTC-ATC-GCA-TTC-3^′^	
	238	Right primer: 5^′^-GTG-CAG-GGT-TGA-CTC-TTT-CTG-3^′^	192255
*cdyl*		Left primer: 5^′^-CTA-CCT-AAG-AGC-ACT-CAC-CTG-AAG-3^′^	
	344	Right primer: 5^′^-ACA-CTT-GAT-CGG-GAT-CTG-AGAC-3^′^	361237
*brdt*		Left primer: 5^′^-CTC-AAG-CTG-CCT-GAC-TAT-TAC-ACC-3^′^	
	382	Right primer: 5^′^-GAG-AGA-GAC-AGA-CAT-CAG-GAA-ACC-3^′^	XM_573544
*hdac1*		Left primer: 5^′^-GAT-GTT-TCA-GCC-TAG-TGC-AGTG-3^′^	
	162	Right primer: 5^′^-GTA-TAG-CCA-TCT-CCT-CCC-AACA-3^′^	AF321129
*miwiI/*		Left primer: 5^′^-GAG-AGG-TTA-CAA-CCC-AAG-ACT-GAC-3^′^	
*piwiLi1*	400	Right primer: 5^′^-GAG-GTA-GTA-GAG-ACG-GTT-GGA-CAG-3^′^	363912
*mbd2*		Left primer: 5^′^-GGC-AAG-AGC-GAT-GTC-TAC-TAC-TTC-3^′^	
	357	Right primer: 5^′^-GCT-AAG-TCC-TTG-TAG-CCT-CTT-CTC-3^′^	680172
*pem(rhoX)*		Left primer: 5^′^-GAG-TGT-CAA-GTC-TGA-GGA-TAA-GCA-3^′^	
	187	Right primer:5^′^-GTA-TGC-AGC-CCT-CCT-AAT-CTT-AAA-3^′^	NM_022175
*cremΔC-G*		Left primer: 5^′^-AGT-CTG-TAC-AGT-CCC-CAG-CAA-CTA-3^′^	
*variant*	322	Right primer: 5^′^-GCA-AGG-GTT-AAG-AGA-CCC-ATC-TAC-3^′^	U048351
*h2a somatic*		Left primer: 5^′^-GAA-TAC-CTG-ACT-GCT-GAG-ATC-CTG-3^′^	
	320	Right primer: 5^′^-ACA-GGC-TCA-GTG-TAC-AGC-ACT-TC-3^′^	64646
*h2b somatic*		Left primer: 5^′^-AGA-AGA-AGG-ACG-GCA-AGG-AAC-3^′^	
	113	Right primer: 5^′^-CAT-GGC-CTT-GGA-AGA-GAT-TC-3^′^	64647
*h3 somatic*		Left primer: 5^′^-TCT-CAT-GAT-GCA-TGT-TTC-TGT-ATG-3^′^	
	345	Right primer: 5^′^-CAC-ACA-CCT-TAA-TGA-CAA-GAC-TCC-3^′^	117056
*th2a*		Left primer: 5^′^-CTC-TTT-CAG-AGC-AGG-TTT-GCAG-3^′^	
	320	Right primer: 5^′^-ACT-TGG-TCT-GGG-ACT-TGT-GGT-3^′^	24828
*th2b*		Left primer: 5^′^-AGT-CAC-CAA-GAC-CCA-GAA-GAAG-3^′^	
	113	Right primer: 5^′^-CTC-GAA-GAT-GTC-TGT-CAC-GAAG-3^′^	24829
*h3t*		Left primer: 5^′^-ACG-TGA-AAT-CGC-TCA-GGA-CT-3^′^	
	196	Right primer: 5^′^-TTA-TGC-TCT-CTC-CCC-TCG-AA-3^′^	682330
*h4*		Left primer: 5^′^-AGG-AGC-CCA-GAG-TTT-GTT-ACAC-3^′^	
	399	Right primer : 5^′^-CTG-GGA-GCA-ATC-TAG-AAG-GATG-3^′^	64627
*Mouse β actin*		Left primer: 5^′^-CTG-GCA-CCA-CAC-CTT-CTA-3^′^	
	238	Right primer: 5^′^-GGG-CAC-AGT-GTG-GGT-GAC-3^′^	007393(44)

## Competing interests

The authors declare that they have no competing interests.

## Authors’ contributions

MKGS conceived and designed study, designed primers, performed TUNEL assay, carried out data analysis, and wrote the manuscript. JC carried out the biological sample collection and immunoassays. MAA carried out the molecular genetic studies. SD carried out electron microscopy studies. All authors read and approved the final manuscript.
